# A theoretical model reveals specialized synaptic depressions and temporal frequency tuning in retinal parallel channels

**DOI:** 10.3389/fncom.2022.1034446

**Published:** 2022-11-16

**Authors:** Liuyuan He, Yutao He, Lei Ma, Tiejun Huang

**Affiliations:** ^1^National Engineering Research Center of Visual Technology, School of Computer Science, Peking University, Beijing, China; ^2^National Biomedical Imaging Center, Peking University, Beijing, China; ^3^Institute for Artificial Intelligence, Peking University, Beijing, China; ^4^Beijing Academy of Artificial Intelligence, Beijing, China

**Keywords:** visual system, retinal adaptation, synaptic depression, retinal circuit, computational modeling

## Abstract

In the Outer Plexiform Layer of a retina, a cone pedicle provides synaptic inputs for multiple cone bipolar cell (CBC) subtypes so that each subtype formats a parallelized processing channel to filter visual features from the environment. Due to the diversity of short-term depressions among cone-CBC contacts, these channels have different temporal frequency tunings. Here, we propose a theoretical model based on the hierarchy Linear-Nonlinear-Synapse framework to link the synaptic depression and the neural activities of the cone-CBC circuit. The model successfully captures various frequency tunings of subtype-specialized channels and infers synaptic depression recovery time constants inside circuits. Furthermore, the model can predict frequency-tuning behaviors based on synaptic activities. With the prediction of region-specialized UV cone parallel channels, we suggest the acute zone in the zebrafish retina supports detecting light-off events at high temporal frequencies.

## Introduction

Our brain processes visual signals in parallel so that we can fast perceive the surrounding environment. This parallel processing begins at the first synaptic layer of the retina, the Outer Plexiform Layer (OPL) (Wässle, 2004; Masland, 2012). Before the OPL, visual signals are encoded by 3∼5 types of photoreceptors (cones and rods) that convert specific wavelengths of light into the membrane potential of the cell body (Baden et al., 2020). Following synaptic transmissions at the OPL, each cone bipolar cell (CBC) subtype forms a specific processing channel that selectively responds to preferred chromatic, spatial, and temporal features in visual signals (Euler et al., 2014; Ichinose et al., 2014; Ichinose and Hellmer, 2016). Understanding how cone synapses distribute visual signals into different CBC channels is a necessary step in the search for parallel processing in the visual neural system.

Ribbon-type synaptic terminals of cones use large proteinaceous structures to hold vesicles behind release sites so that synapses can preciously encode graded changes in membrane potential into continuous glutamate release (Lagnado and Schmitz, 2015; Moser et al., 2020). Although synaptic ribbons accelerate the vesicle resupply rate, the large number of vesicles required by high membrane potentials (>−35 mV) cannot be met (Van Hook et al., 2014; Maxeiner et al., 2016; Mesnard et al., 2022). Thus, a high membrane potential might initially trigger a high release rate in cone terminals, but the release rate decreases over time (Jackman et al., 2009). To access the property of synaptic depression, paired-pulse depression (PPD) experiments use the 1st pulse with a high membrane potential to trigger a peak response and deplete restored vesicles. Then, it uses a second pulse to measure the depression degree and estimate the recovery time constant from depression (Singer and Diamond, 2006; Vickers et al., 2012; Grabner et al., 2016).

Several experiments have observed different depression recovery time constants at postsynaptic cells among parallel CBC channels (DeVries, 2000; DeVries et al., 2006; Grabner et al., 2016). Both the synaptic structure and molecular mechanisms contribute to such diversity. The cone contains a unique structure, an invagination of the plasma membrane, which forms unequal postsynaptic contacts with different travel distances for neurotransmitters (Haverkamp et al., 2000; Behrens et al., 2016). Recent studies have shown that invagination contacts have relatively smaller recovery time constants due to the saturation of postsynaptic receptors (Grabner et al., 2016). Besides, the vesicle movement properties and calcium dynamics at presynaptic terminals also vary among cones (Grassmeyer and Thoreson, 2017; Schroeder et al., 2021). Furthermore, the neurotransmitter receptors at CBC dendrites differ even within subtypes with the same polarity (DeVries, 2000; Lindstrom et al., 2014). In conclusion, depressions at the cone-CBC synapses are specialized for the CBC and cone subtypes.

**FIGURE 1:**
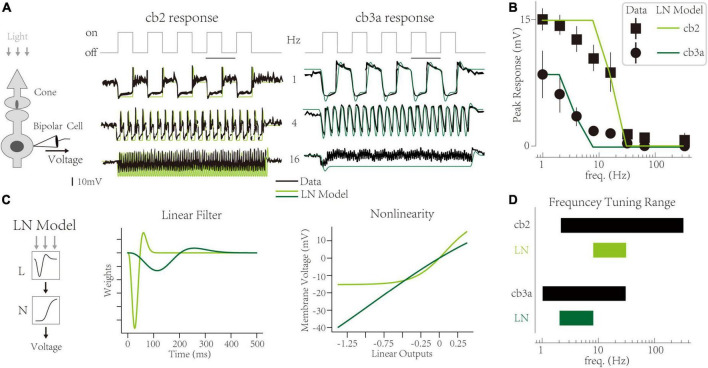
Temporal frequency tuning of cone bipolar cell channels. **(A)** The membrane voltage responses of retinal cone bipolar cells (CBCs) that adapt to temporal frequencies in lights. (Left) The experimental protocol involves the neural circuit with a cone, a CBC, and the ribbon synapse contact with two neurons. (Center and Right) Responses of the cb2 and the cb3a subtype in experimental recordings [black, adapted from Grabner et al. (2016)] and simulated traces from Linear-Nonlinear (LN) models (green and seaweed). **(B)** The temporal frequency tuning with peak responses relative to the resting potential of two subtypes. Linear-Nonlinear (LN) models’ responses (green and seaweed) are inconsistent with recordings [black, adapted from Grabner et al. (2016)]. **(C)** The LN model represents two circuits’ neural computations. (Left) Schematic of the LN model for two subtypes. (Center) Linear filters. (Right) Non-linearities. **(D)** The frequency tuning range of biological cells (black) and LN models (green and seaweed).

At the functional level, synaptic depression limits the amplitude of CBC responses at high frequencies. Take off-type CBCs as an example. A light-dark switch triggers a transient response of off-type CBCs, mainly because cone terminals store vesicles in the light and deplete vesicles in the dark (Jackman et al., 2009; Maxeiner et al., 2016). The peak amplitude of transient responses is evident at low frequencies (1 HZ). With the increment of temporal frequency, the transient response decreases (Figure 1A) [adapted from Grabner et al. (2016), also observed in Ichinose and Hellmer (2016)]. Two subtypes (cb2 and cb3a) share the same cone terminal but are distinctly sensitive to temporal frequencies (Figure 1B). The experiment suggests that such temporal frequency tunings are mainly due to the limitation of synaptic depression (Grabner et al., 2016). It indicates that synaptic contact with a larger recovery time constant is more sensitive to high temporal frequencies. However, the linkage between synaptic-level activities and circuit-level functions is not quantified. It is unclear whether we can infer the synaptic depression from temporal tuning behaviors or predict the temporal tuning based on synaptic plasticity.

This work quantifies the link between synaptic depressions and temporal frequency tunings among type-specialized and region-specialized retinal channels. We use the hierarchy Linear-Nonlinear-Synapse (hLNS) framework, a flexible tool to build the retinal circuit model with cell and synaptic blocks (He et al., 2022). It has an inner variable to represent the membrane voltage of neurons so that we can apply circuit-level and synapse-level experiments on circuit models. Furthermore, the hLNS framework has a kinetics block to infer the synaptic depression in circuits and robustly estimate the depression recovery time constant in related synapses. It has successfully inferred the adaptive properties of retinal ribbon synapses from various experimental recordings, including the vesicle release traces and the postsynaptic recordings at both membrane potential and light stimuli (He et al., 2022).

We first build a Linear-Nonlinear-Synapse (LNS) model for the cone-CBC circuit. The LNS model uncovers experiment-validated short-term depressions of cone-CBC synapses from the CBC-type-specialized membrane potential responses under different temporal frequencies (Grabner et al., 2016). Then, we use it to capture synaptic activities among region-specialized UV cones in the zebrafish retina (Schroeder et al., 2021). Furthermore, we predict that the UV cone synapses in the acute zone (AZ), the region with the highest visual acuity that helps the zebrafish capture prey, produce transient responses at higher frequencies (∼10 HZ). In contrast, responses in other regions are disappeared. In conclusion, our works give a new theoretical approach to understanding cone synapses’ role among parallel retinal processing channels.

## Results

### The Linear Nonlinear Synapse model captures type-specialized frequency tunings

Previous electrophysiological experiments usually record membrane voltages of retinal CBCs to illustrate the temporal frequency tuning curve of processing channels but ignore synaptic depressions inside the circuit (Burkhardt et al., 2007; Ichinose et al., 2014; Ichinose and Hellmer, 2016). In this paper, we found an experiment recording both membrane potentials with varied temporal frequency and synaptic depressions in CBCs with two subtypes (Grabner et al., 2016). Thus, we can validate our theoretical model by capturing the temporal frequency tuning of CBCs and testing whether it successfully infers synaptic depressions. In this section, we first use the LN model to highlight the importance of synaptic adaptations among two CBC-type-specialized channels. We then show that our theoretical models can capture different frequency tunings among cone-CBC channels.

In a ground squirrel retina, two subtypes (cb2 and cb3a) of off-type CBCs have different frequency tunings [Figure 1A, adopted from Grabner et al. (2016)]. The interaction between two channels forms critical retinal functions, typically direction selection (Kim et al., 2014; Ray et al., 2018). At a low frequency (1 Hz), the membrane potential of two CBCs fluctuates after the light’s turn-on. It is a typical response of off-type retinal cells and has also been observed in other experiments (Jackman et al., 2009; Odermatt et al., 2012). Following the original paper, we use the “peak response,” the max voltage amplitude relative to the dark baseline level at the initial, as the feature of neural responses at a given frequency. With the increment of frequency, peak responses of two subtypes go their separate ways and eventually disappear at a maximum frequency (333 Hz) (Figure 1B). Such differences are evident in traces at the 4 Hz, where cb2 cells keep their peak responses while cb3a cells lose >50% peak responses. Ant at 16 Hz, cb2 cells still have ∼50% responses while cb3a cells’ peak responses are weak. The task of a computational model is to capture these peak responses in two distinct subtypes.

The Linear-Nonlinear (LN) model uses two abstract components, a linear temporal filter and a static non-linearity (Baccus and Meister, 2002), to explain the differences between the two subtypes (Figure 1C left). It found that the cb3a cell has a slower temporal filter (∼300 ms) than the cb2 one (∼100 ms). Moreover, the effects of the nonlinear transform in the cb3a are minimal. In contrast, the cb2 cell needs a non-linearity with a threshold to tune the fluctuation of the membrane voltage. The LN model of both subtypes produces the disappearance of peak responses at high frequencies. However, the LN model cannot deftly capture exact peak responses at middle temporal frequencies. For example, the LN model for cb2 cells keeps high peak responses at 8 Hz, while natural cells only remain at ∼70% peak responses. Moreover, the LN model for cb3a cells loses the peak response at 8 Hz, while the natural ones still have ∼20% peak responses. To illustrate the frequency tunning features, we define a cell’s tuning range that begins at the highest frequency without decreasements and ends at the minor frequency when the transient responses disappear. In both subtypes, the tuning range of the LN model is smaller than the biological cell, suggesting that the LN model cannot capture the sensitivity to temporal frequencies (Figure 1D). Therefore, the LN model fails to capture CBC subtypes’ precise frequency tunings.

**FIGURE 2:**
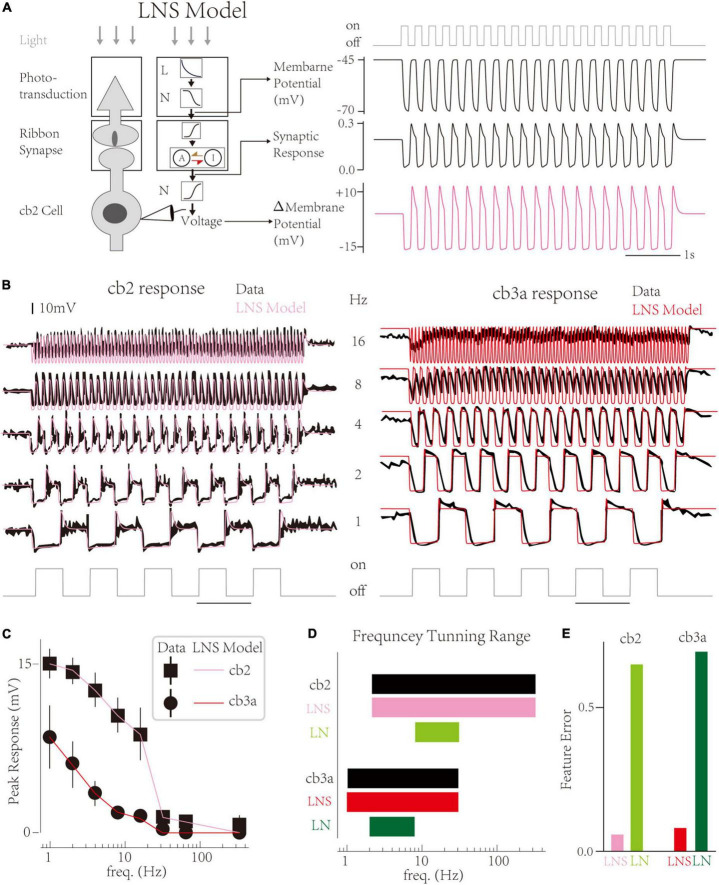
The Linear-Nonlinear-Synapse model captures temporal tunings of two cone bipolar cell (CBC) channels. **(A)** Schematic of the Linear-Nonlinear-Synapse (LNS) model (Left) The LNS model has three stages to mimic the cone-CBC circuit [adapted from He et al. (2022)]. (Right) Three stages’ outputs have biological meanings. At the 4 Hz light trace, the LNS model yields the membrane potential of the cone, the synaptic response of the cone-cb2 contact, and the membrane potential of the cb2 cell relative to the resting potential. **(B)** Simulated traces of the LNS model (pink and red) overlaid with experimental recordings (black). **(C)** The temporal frequency tuning with peak responses of LNS models are consistent with recordings. **(D)** The frequency tuning range of LNS models (pink and red) and LN models (green and seaweed). **(E)** The performance of the LNS and LN models after optimization.

In this work, we propose a LNS model that contains a specific block to highlight the function of synapses in CBC responses and improve biological interpretability (Figure 2A left; He et al., 2022). In the LNS model, the light stimuli are turned into the cone membrane potential by an LN block and then transformed into the synaptic response through a synaptic block. The synaptic block uses the synapse’s Calcium Voltage-Current function to convert the cone membrane voltage into the driving force to control a kinetics system named the RAB module (Figure 2A left middle). States and rate constants in the RAB module are defined as


AActive    αResponse(A→I)

IInactive    βRecovery(I→A)

And the output of the synaptic block is the transfer rate from the active state to the inactive state (see Section “Materials and Methods”). Furthermore, a non-linearity follows the synaptic block to produce the membrane potential of CBCs. Therefore, inner variables before and after the synaptic block have specialized biophysical meanings, the cone membrane voltage, and EPSCs at CBC dendrite correspondingly (Figure 2A right).

Results show that the LNS model successfully captures the temporal tunning of two cells (Figure 2B). Overall, the LNS models’ decrease curves of peak responses perfectly match the experimental recordings (Figure 2C). Compared to the LN model, the peak response of the LNS model for the cb2 subtype slightly decreases until 8 Hz. In addition, the LNS model for the cb3a subtype keeps small peaks around 8 and 16 Hz, matching the experimental measurements. Altogether, the frequency tuning curves of the LNS models for two subtypes are identical to biological cells (Figure 2D). By comprising response features, we found that the performance of the LNS model is much better than the LN model (Figure 2E). In conclusion, the LNS model successfully captures the temporal frequency tuning on CBC channels.

### The Linear-Nonlinear-Synapse model successfully infers type-specific synaptic depressions

The original experimental paper suggests that the ribbon synapse contacting the cone pedicle and the CBC dendrite limits peak responses at high frequencies (Grabner et al., 2016). In detail, differences in depression recovery time constants among synaptic contacts produce varied temporal tuning phenomena. Notably, the LNS model highlights the contact between two cells and inherits the theoretical analysis approach of synaptic depressions from the hLNS framework (He et al., 2022). Therefore, the synaptic depression properties inside LNS models must match experimental measurements. In this section, we first show the theoretical analysis results of synaptic depression time constants of LNS models for two channels. Then, we apply the PPD experiment to the LNS model to test whether simulated synaptic behaviors are consistent with experimental recordings.

**FIGURE 3:**
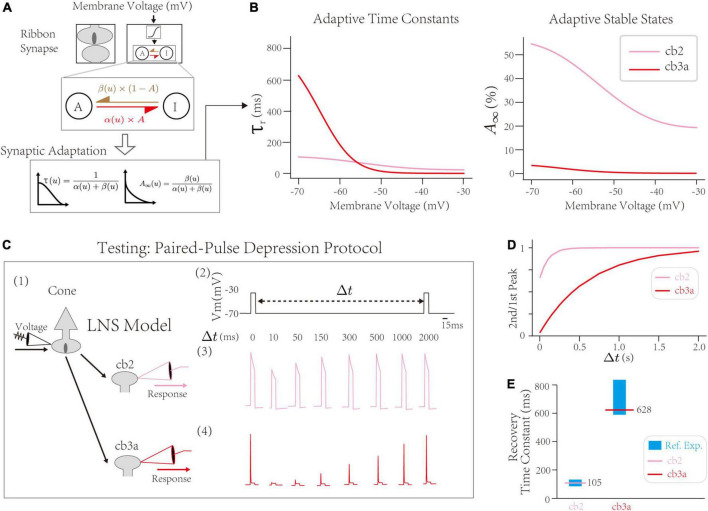
The validation of synaptic depressions inside the Linear-Nonlinear-Synapse (LNS) model. **(A)** The synaptic block inside the LNS model provides a theoretical inference of synaptic depressions, adapted from He et al. (2022). **(B)** Prosperities of synaptic adaptations inferred by the LNS model. (Left) The adaptive time constant and (Right) stable state alongside the cone membrane potential. **(C)** Testing the synaptic depression inside the LNS model with the Paired-Pulse Depression (PPD) experiment. (1) The experimental protocol that manipulates the input for the synaptic block, *V*_*cone*_, and records the outputs of the synaptic block, *r*. It mimics the experiment that changes the cone potential and records EPSCs at the CBC dendrites. (2) A sample input trace for the PPD protocol. Two pluses (–30 mV and lasts 10 ms) are triggered in the presynaptic potential, gap by a time interval Δ*t*. (3 and 4) the outputs of the synaptic block in the LNS model for the cb2 subtype (3) and the cb3a subtype (4). **(D)** The recovery process (the 2nd/1st pulse peak) in two LNS models. **(E)** Recovery time constants of two LNS models are consistent with reference experimental recordings. In the cb2 LNS model, 105 ms vs. 125.7 ± 9.6 ms. In the cb3a LNS model, 628 ms vs. 698 ± 132 ms.

First, the synaptic block infers cone-CBC contact’s recovery time constant at the PPD protocol. Inside the synaptic block, the RAB module with the active-inactive transfer system generates adaptive responses of synapses. The intrinsic dynamics of the synaptic block are determined by two transfer rate constants, α and β, between active/inactive states (Figure 3A up). The theoretical analysis yields two adaptive properties, the adaptive time constant τ_*r*_, indicating the speed of the synaptic adaptation at given input, and the stable state *A*_∞_, indicating the steady-state if the active state A mentioned in the Section “The Linear Nonlinear Synapse model captures type-specialized frequency tunings” [Figure 3A down, see Section “Materials and Methods,” and the original theoretical analysis in He et al. (2022)]. Precisely, τ_*r*_ at −70∼−60 mV indicates the depression recovery time constant measured by the PPD protocol, where the synaptic recovery occurs at hyperpolarized voltages (Burrone and Lagnado, 2000; Rabl et al., 2006; Singer and Diamond, 2006; Li et al., 2007; Grabner et al., 2016). Results suggest the recovery time constant of the cone-cb2 synapse is ∼100 ms, less than the recovery time constant of the cone-cb3a synapse (∼600 ms). Therefore, the recovery of the cone-cb2 synapse is faster than the cone-cb3a one, inferred by the LNS model.

Next, we introduce the PPD protocol with experimental recordings to validate short-term depressions inferred by the LNS model. The PPD protocol from Grabner et al. (2016) holds the cone at −70 mV at resting except for pulses that raise the voltage to −30 mV (Figure 3C). We directly apply the voltage trace as the input for the synaptic block and record the outputs of the block analogous to EPSCs. The PPD protocol triggers a fast recovery process (τ = 105 ms) of the short-term depression in cone-cb2 synapses and relatively slow recovery (τ = 628 ms) in the cone-cb3a synapse (Figure 3D). Both time constants from the LNS models are within the range measured by experiments (Figure 3E). Therefore, the LNS model successfully uncovers the recovery time constant of the cone-CBC synapse from the experimental recordings about temporal filtering on CBC responses.

In conclusion, our LNS model found that the depression recovery in the cone-cb3a synapse is slower than in the con3-cb2 synapse, consistent with the experimental measurements on two synapses.

### Inner dynamics inside the frequency tuning of the cone bipolar cell channels

In this section, we use the LNS model to understand how synaptic depression contributes to temporal frequency tuning in retinal channels. We first explain why the LNS model produces a peak response at 1 Hz and depresses its peak responses at high frequencies. Then, we found the link between the recovery time constant and the inner dynamics of the synaptic block, determining the CBC channel’s sensitivity to frequencies.

**FIGURE 4:**
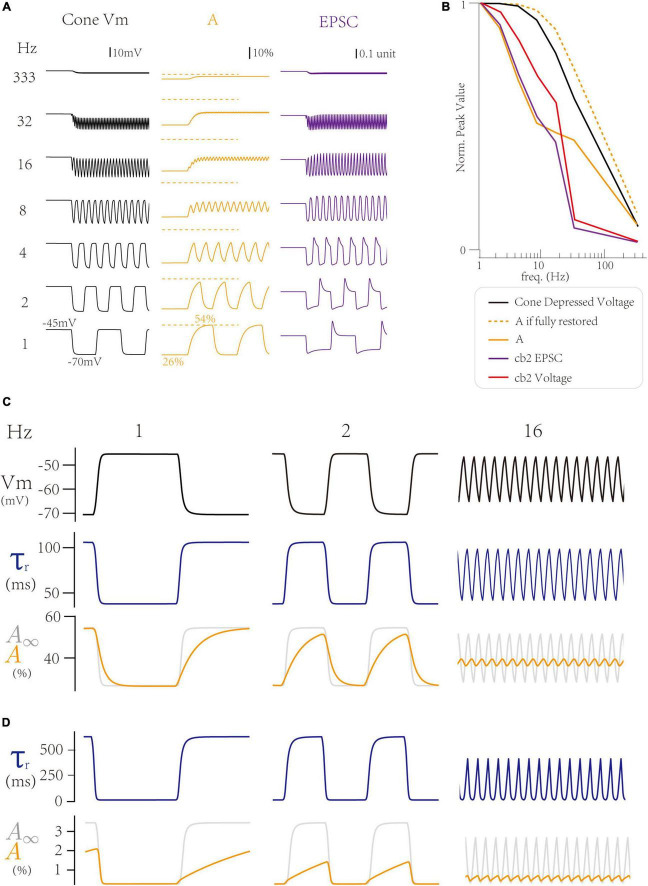
Inner dynamics of the Linear-Nonlinear-Synapse (LNS) model. **(A)** The inner variables of the cb2 LNS model change with frequencies. (Left) The cone membrane potential. (Center) The active state A inside the synaptic block. (Right) The synaptic responses (EPSCs). **(B)** The peak values of inner variables of the cb2 LNS model. **(C,D)** Adaptive time constants change the recovery process of two circuits. From up to down, the cone potential, the time constant, and the active state relative to the stable state in the cb2 LNS model **(C)** and the cb3a LNS model **(D)**.

First, we explore how the LNS model produces a peak response at 1 Hz, the baseline frequency. Notably, both the temporal filter in the cell block and the synaptic bock affect the frequency tuning curve. At 1 Hz, where the peak response is maximum, the cone membrane potential goes to the most hyperpolarized voltage (−70 mV) at the off step (Figure 4A left). In the LNS model for the cb2 CBC, the change of cone membrane potential from −70 to −45 mV produces an adaptive process in the synaptic block, whose active state A decreases to 26% in the light and increases to 54% in the dark (Figure 4A center). Therefore, the on-off switch triggers a peak response in the synaptic responses by raising the cone membrane potential and losing restored active states (Figure 4A, right). In conclusion, the trigger of a peak response needs a hyperpolarized voltage in the cone membrane potential and a time interval at the light that enables the RAB module to adapt to its high steady-state of the active state A.

**FIGURE 5:**
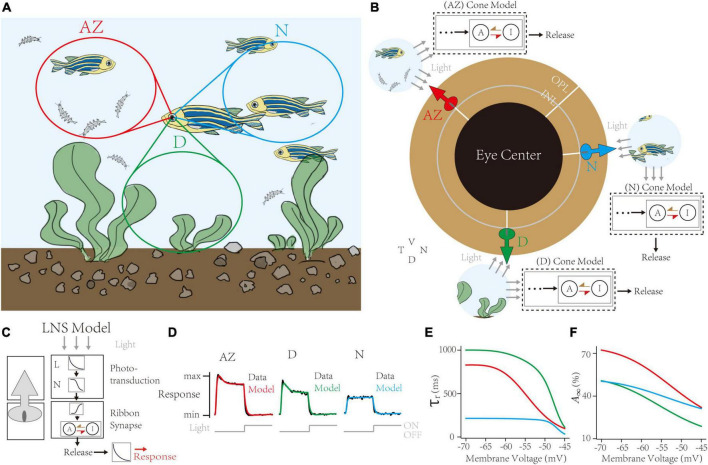
Capturing synaptic adaptations in region-specialized zebrafish UV cone synapses. **(A)** In the retina of a swimming zebrafish, three regions process distinct inputs from the visual field. AZ for Acute Zone, to detect and capture prey. D for dorsal, for the downsides. N for nasal, for the outward. **(B)** The single region has its cone model to infer the dynamic adaptation in ribbon synapses. **(C)** Schematic of the Linear-Nonlinear-Synapse (LNS) model for UV cone vesicle releasement. **(D)** Response curves of models and datasets in three regions. Dark lines for experimental datasets [adapted from Schroeder et al. (2021)]. Colored lines for models in corresponding regions. **(E,F)** Inferred synaptic adaptations in LNS models for the adaptive time constant **(E)** and the stable activate state **(F)**.

Next, we investigate the inner dynamics of the LNS model of the cb2 CBC to find the underlying mechanisms that limit peak responses. The hyperpolarized voltage majorly varies the response at very high frequencies (>30 Hz), where the cone membrane potential stays around −45 mV due to a larger time constant (40 ms) in the temporal filter of the LN block. Therefore, the peak response disappears at high frequencies (Figure 5B, black line). In contrast, at middle frequencies (4∼16 Hz), the synaptic depression makes significant contributions. These frequencies shorten the time interval at lights so that the active state A (Figure 4B, yellow line) cannot fully recover to its theoretical maximum values *A*_∞_ (Figure 4B, yellow dash line). Therefore, the response is depressed due to the depletion of the active state A. In conclusion, the frequency temporal tunning at 1∼16 Hz is from the synaptic depression between the cone and the CBC dendrite.

Following the above analysis, we grasp why the cb3a subtype is more sensitive to the increase of the temporal frequency than the cb2 subtype. As the cone-cb3a contact has a larger recovery time constant, restoring to its steady-state requires a longer time. Therefore, at 1 Hz, the time interval at dark is 500 ms, which is insufficient for the cone-cb3a synapse to recover (Figure 4D, left). Such depression is more significant at 2 Hz, where the cone-cb3a synapse slightly recovered (Figure 4D, center) while the cone-cb2 synapse almost fully recovered (Figure 4C, center). Therefore, the slow recovery time constant in the cone-cb3a synapse enable the cb3a CBC to change its peak response at relatively low frequencies (2∼8 Hz) (Grabner et al., 2016).

### Predict the temporal tunning of location-specialized retinal channels

This work shows that the LNS model connects the short-term depression in the cone-CBC synapse and the temporal frequency tuning in the retinal visual channel. Here, we investigate the temporal tuning from another side: if we infer synaptic depressions from experimental datasets, can we predict the differences in temporal frequency tunings among parallel channels? In this section, through a set of response traces of zebrafish cones, we theoretically infer synaptic depressions in zebrafish UV cones depending on their locations in the eye (Schroeder et al., 2021). Furthermore, we predict that cones in the AZ support the transmission of visual signals at around 16 Hz. We suggest that highly specialized visual channels are the outcome of acclimatizing to the living environment.

The retina tissue in a swimming zebrafish is divided into heterogeneous regions receiving input signals (Figure 5A; Zimmermann et al., 2018; Yoshimatsu et al., 2020). These regions are categorized into two groups: the AZ, having peak UV cone density to precept and prey UV-bright water-borne microorganisms, and the Non-AZ regions, including the nasal (N) and the dorsal (D) region, whose UV cone densities are smaller, and cell responses are weaker (data lines in Figure 5D). Notably, UV cones in the AZ and dorsal regions have transient responses when the light turns off. We use the LNS model for each region to capture location-specialized UV-cone release traces (Figure 5B). Specifically, the outputs of the synaptic block in the LNS model are the presynaptic vesicle releases instead of EPSCs. Besides, to simulate the iGluSnFR recording protocol, we added a kernel to convolve response traces (Figure 5C). We used corresponding de-noised average responses of cones under the light stimulus in three regions to train separate models. Results show the high accuracy of fittings on all channels (correlation >0.99, Figure 5D).

**FIGURE 6:**
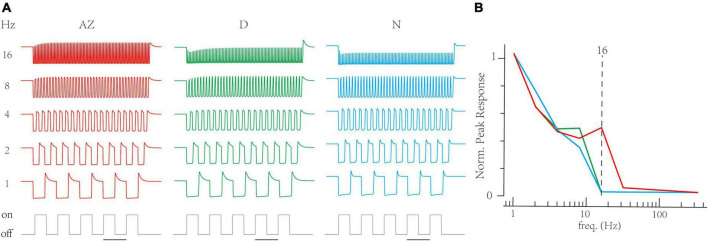
The Linear-Nonlinear-Synapse (LNS) model predicts that UV cones in the acute zone support high-frequency signals. **(A)** The transient responses of cones are depressed when stimulus frequency is up. The below trace is the stimulus timing for the 1 Hz trace (bar for 1 s). Frequencies are at the right. **(B)** The relationship between transient responses and frequencies in three region-specialized cones. The peak response is the gap between the maximum response in the last 1 s and the resting response before simulation. The Dash line marks responses at 16 Hz.

Next, the RAB module in models illustrated short-term depressions in three channels (Figure 5E). Compared to non-AZ regions, AZ cones have a marked higher steady-state value at −70 mV (Figure 5F). It suggests AZ cones hold more releasable vesicles, which is consistent with the volumetric electron microscopy datasets in the original paper (Schroeder et al., 2021). On the contrary, AZ cones have similar recovery time constants at the hyperpolarized potential to dorsal cones. Based on optimized LNS models, we predict the temporal frequency tuning of location-specialized channels (Figure 6). We stimulate cone models with frequency-varied light stimuli (Figure 6A) and gather the relationship between peak responses (vesicle release rates) and frequencies from 1 to 333 Hz (Figure 6B). Results show that under the 16 Hz light stimulus, the cone in the AZ holds high peaks above the resting response. In contrast, peak responses in non-AZ ones are disappeared. It indicates that UV cone synapses in the AZ can respond to light-off events at relatively high frequencies. The support for event detections might play a fatal role in accurately capturing microorganisms.

In conclusion, we found that UV cones support high-frequency signals’ transmission, suggesting that visual channels in this location help zebrafish encode fast-changing visual features and live in an immediate and complicated environment.

## Discussion

The cone pedicle in a vertebrate retina is supposed to be the most complicated synapse in the central nervous system (Haverkamp et al., 2000). A cone pedicle reliably delivers signals into ∼2 horizontal cells and ∼8 bipolar cells, using 20∼50 presynaptic active zones and ∼500 contacts with downstream neurons (Wässle, 2004; Matthews and Fuchs, 2010). Unequal connections between the cone pedicle and downstream neurons are first identified in DeVries (2000), suggesting that different glutamate receptors produce distinct signals on postsynaptic dendrites. With advanced techniques, the diversity of molecules (Borghuis et al., 2014; Lindstrom et al., 2014) and electrophysiological properties (DeVries et al., 2006; Grabner et al., 2016) are identified among cone-CBC contacts, supporting that the cone pedicle filters visual information into location-specialized and subtype-specified channels. As a typical sensory synapse (Fortune and Rose, 2001), the cone synapse contributes to low-pass filterings, enabling each BC subtype to exhibit a unique temporal profile in its membrane potentials and synaptic outputs, representing an elementary building block in retinal circuits (Ichinose et al., 2014; Ichinose and Hellmer, 2016). In this work, we include the synaptic activity and the neural responses into a theoretical framework, the LNS model, that can reliably predict synaptic depressions from neural temporal filtering (Figure 3; Grabner et al., 2016). Conversely, it predicts biologically reasonable temporal filtering from synaptic depressions (Figure 6). Therefore, our study has illustrated how cone synapses form parallel channels that process temporal visual signals from the environment.

Understanding synaptic activities from sensory neurons’ responses is a traditional issue in computational neuroscience. The standard LN model uses two statistic components, a linear spatial-temporal filter and a nonlinear transform, to abstract the processing of retinal neurons. Due to their statistics, a single LN model poorly captures the neural activities at varied frequencies and contrasts (Baccus and Meister, 2002; Jarsky et al., 2011). Several works appended a block behind an LN model to grab the function of ribbon synapses and neural responses at lights, such as (Baden et al., 2014; Schröder et al., 2019; Schroeder et al., 2021). Among these works, the hLNS framework is the only one that satisfies two requirements in this work (He et al., 2022). First, it has an inner variable to simulate the membrane voltage of neurons so we can apply the PPD experiment to validate the synaptic depression inside the model. In contrast, we cannot use models whose LN blocks convert the lights into calcium signals (Ozuysal et al., 2018; Schröder et al., 2019, 2020) and abstract models without synaptic blocks (Ozuysal and Baccus, 2012; Heitman et al., 2016). Second, the synaptic block is a general and abstract model as two experimental recording datasets involve different underlying mechanisms. However, most synaptic blocks in circuit models mimicking the cascade of vesicle pools are particular for vesicle-related mechanisms (Baden et al., 2014; Schroeder et al., 2021). Consequently, these blocks are unsuitable for obtaining mechanisms from postsynaptic dendrites in CBC channels and calcium dynamics in cone channels. Instead, the RAB module in the hLNS framework is an abstract block that successfully covers mechanisms besides vesicle movements and has high robustness for unseen stimuli that trigger synaptic adaptations. Therefore, to capture the synaptic depression inside circuit responses, we suggest the theoretical model needs to explicitly state the cone membrane potential and use a synaptic block with a high ability of generalization and robustness.

The frequency tuning in the LNS model is the outcome of both the LN block, representing the function of the phototransduction, and the RAB module, describing the role of ribbon synapses (Figure 4). In general, phototransduction also might contribute to the diversity of parallel processing channels. Recent studies suggest that multiple subtypes of cones differ in intrinsic phototransduction, so cone somas yield type-specific frequency tunings (Baudin et al., 2019). In this work, we unify the phototransduction process among parallel channels into an identical LN block, as anatomical results suggest that the cb2 and the cb3a channels share the same cone subtypes in the ground squirrel (DeVries et al., 2006) and the mouse retina (Behrens et al., 2016). In the experimental recordings of subtype-specialized CBC channels, synaptic depression results from multiple mechanisms, the presynaptic vesicle depletion, the unequal structures for invaginating/non-invaginating contacts, and the kinetics of postsynaptic receptors (DeVries, 2000; DeVries et al., 2006; Lindstrom et al., 2014; Behrens et al., 2016; Grabner et al., 2016). In contrast, the experimental recordings of region-specialized UV cone channels involve presynaptic mechanisms, mainly vesicle movements and calcium-related dynamics (Schroeder et al., 2021).

In the LNS model, neural activities outside the cone synapse are replaced by linear filters and non-linearities. Therefore, the LNS model ignores several fatal neural dynamics inside the circuit that might contribute to the adaptive activities. One crucial source of adaptations is phototransduction (Burns and Baylor, 2001; Dunn et al., 2007), whose adaptive processes are beyond a static LN model (Clark et al., 2013). Another is the dynamics of ion channels alongside the surface of CBCs, such as the calcium (Hu et al., 2009), sodium (Zenisek et al., 2001), potassium (Hu and Pan, 2002), and HCN channels (Ivanova and Mueller, 2006). These ion channels are supposed to form transient responses of retinal bipolar cells (Baden et al., 2011; Saszik and DeVries, 2012), which appear under pulses at the PPD protocol (Figure 3). Therefore, a more biophysical model combining both synapses and other mechanisms is necessary for a deeper understanding of the role of synaptic depressions in frequency tuning.

In the case of the zebrafish retina, the original experiment (Schroeder et al., 2021) and related works (Zimmermann et al., 2018; Yoshimatsu et al., 2020) have illustrated regional differences between the AZ and other zones, including the phototransduction, neural anatomy, synaptic calcium signal, glutamate releases, and interactions with horizontal cells. In this work, the LNS model suggests that UV cones in the AZ can detect the light-off events at relatively high temporal frequencies where ones in other regions failed to produce peak responses (Figure 6). Although biological experiments haven’t directly validated these predictions, they are consistent with previous experimental and theoretical discoveries. In Yoshimatsu et al. (2020), the glutamate imaging experiments found that only synaptic releases of UV cones in the AZ responded strongly to light-off events with the small time intervals, while their temporal frequencies (the binary sequence at 12.8 Hz) are smaller than the one (16 Hz) in our predictions (Figure 6B). Furthermore, Schroeder et al. (2021) proposed a computational model for UV cone channels and predicted that the UV cones in the AZ region support the detection of light-on events. Therefore, alongside the history, our predictions are coherent with the principle that cells and circuits in the AZ region boost sense of UV vision so that a zebrafish can capture the presence of prey.

In conclusion, our study provides a theoretical approach to bridging the gap between synaptic depression and frequency tuning of retinal parallel processing channels. We suppose the inference of synaptic depressions from frequency tuning curves and the prediction of frequency tuning from synaptic depressions help us understand how the retina smartly processes the vivid environment.

## Materials and methods

This work used experimental recordings of ground squirrel off-type bipolar cell EPSCs and vesicle release rates of zebrafish UV cones. The experiment on bipolar cells involves two subtypes and provides features of frequency tuning and synaptic adaptations (Grabner et al., 2016). The cone experiment only provides synaptic responses of three location-specialized subtypes (Schroeder et al., 2021). In this section, we first describe a baseline model, the Linear-Nonlinear (LN) model, to highlight the adaptive activities in temporal tunings. Based on this precursor, we describe the hierarchy Linear-Nonlinear-Synapse (hLNS) framework and customized LNS models to infer synaptic depressions from the experimental recordings of the frequency tuning curve. Then, we introduce the stimulus protocol and related recordings in experiments for measuring frequency tuning of retinal neurons and synaptic adaptations of cone-BC synapses. At last, we describe the details of model implementation and optimization.

### The linear nonlinear model

The Linear-Nonlinear (LN) model has been widely used to capture activities and quantify the adaptive properties of retinal cells (Baccus and Meister, 2002). It consists of a linear temporal filter to mimic the receptive field of a retinal neuron and a static non-linearity to tune the response amplitude. In this work, the light stimuli *x*(*t*) are first convolved by the filter kernel *L_LN_*(*t*), yielding the linear outputs *l*(*t*), as


(1) $$l\,\left(t\right)=\int_{-\infty }^{T}x\,\left(t\right)*L_{\mathrm{LN}}\,\left(t-T\right)\,\mathrm{dt}$$

To capture the transient response of bipolar cells, we use the photoreceptor impulse filter for *L_LN_*(*t*), as


(2) $$L_{\mathrm{LN}}\,\left(t\right)=\frac{-{\left(\frac{t}{\gamma \,\tau_{r}}\right)}^{3}}{1+\frac{t}{\gamma \,\tau_{r}}}*\exp\left(-{\left(\frac{t}{\gamma \,\tau_{d}}\right)}^{2}\right)*c\,o\,s\,\left(\frac{2\,\pi \,t}{\gamma \,\phi }+\tau_{\mathrm{phase}}\right)$$

where τ_*r*_ = 70 ms, τ_*d*_ = 70 ms, τ*_phase_* = 100 ms, ϕ = -π/5 are fixed, and γ > 0 is free for optimization (Baden et al., 2014; Schröder et al., 2019). Next, the nonlinear function *N_LN_*(*l*) transforms the linear outputs *l*(*t*) into the membrane potential of bipolar cells *V_LN_*(*t*) relative to the resting potential, as


(3) $$V_{\mathrm{LN}}\left(t\right)=N_{\mathrm{LN}}\left(l\left(t\right)\right)-N_{\mathrm{LN}}\left(l\left(x\left(t\right)=0\right)\right)$$

Here we use a sigmoidal non-linearity to capture the membrane potential of bipolar cells:


(4) $$N_{\mathrm{LN}}\,\left(l\right)=\frac{m}{1+\exp\left(-\frac{l-\mathrm{half}}{\mathrm{slope}}\right)}$$

where *m*, *half*, and *slope* are parameters for optimization.

### The hierarchy Linear-Nonlinear-Synapse framework

The core idea of the hLNS framework is that the neuron in retinal circuits is modeled by a Linear-Nonlinear (LN) block, and an adaptive block highlights the synapse between two neurons (He et al., 2022). The LN block (Equations 1, 3) in the hLNS framework yields the membrane potential *V_cell_*(*l*(*t*)). The synaptic block uses a nonlinearity to transform the membrane voltage *V_cell_* into the driving force *u_cell_* to control the RAB module, which is a two-state kinetics block (Figure 2A, and Section “The Linear Nonlinear Synapse model captures type-specialized frequency tunings”). The output of the RAB module is the transient rate from the active state to the inactive state, as


(5) $$r\,\left(u_{\mathrm{cell}}\,\left(t\right)\right)=\alpha \,\left(u_{\mathrm{cell}}\,\left(t\right)\right)\cdot A\,\left(t\right)\cdot N$$

where N is a free parameter to scale the amplitude of responses, and *A* presents the current percentage of the active state. The synaptic block has two significant adaptive properties, the stable state of the active state *A*_∞_(*V_cell_*) and the time constant τ_*r*_(*Vcell*). They are determined by two rate constants as


(6) $$A_{\infty }\,\left(V_{\mathrm{cell}}\right)=\frac{\beta \,\left(u_{\mathrm{cell}}\,\left(V_{\mathrm{cell}}\right)\right)}{\alpha \,\left(u_{\mathrm{cell}}\,\left(V_{\mathrm{cell}}\right)\right)+\beta \,\left(u_{\mathrm{cell}}\,\left(V_{\mathrm{cell}}\right)\right)}$$

(7) $$\tau_{r}\,\left(V_{\mathrm{cell}}\right)=\frac{1}{\alpha \,\left(u_{\mathrm{cell}}\,\left(V_{\mathrm{cell}}\right)\right)+\beta \,\left(u_{\mathrm{cell}}\,\left(V_{\mathrm{cell}}\right)\right)}$$

### The Linear Nonlinear Synapse models

In this work, the cone-CBC circuit only involves two cells and a single synapse. Our LNS model obtains an LN block and a synaptic block to mimic vesicle releases or postsynaptic currents of cone synapses (Figure 2A).

The LN block in the LNS model represents the phototransduction process that converts light into the membrane potential of the cone *V_cone_*. To ensure the reasonability, this work adapts formulations of the linear filter and the non-linearity from experiments in cones (Baylor et al., 1974). Specifically, the linear filter *L_LN_* (*t*) is


(8) $$L_{\mathrm{LNS}}\,\left(t\right)=e\,x\,p\,\left(-\frac{T-t}{\tau_{L}}\right)$$

where τ_*L*_ = 40 ms, and the non-linearity is


(9) $$V_{\mathrm{cone}}\,\left(t\right)=-26.4*\frac{l^{4.92}}{l^{4.92}+0.542^{4.92}}-45$$

Notably, these formulations and parameters are fixed during optimization.

The synaptic block in the LNS model represents the synaptic transmission or vesicle releasement of cone pedicles. The nonlinear function that transforms the cone membrane voltage *V_cone_* into the driving force *u_cone_* is adapted from the I-V curve of Cav1.4 (Grassmeyer and Thoreson, 2017), as


(10) $$u_{\mathrm{cone}}\,\left(V_{\mathrm{cone}}\,\left(t\right)\right)=0.078*\frac{41-V_{\mathrm{cone}}\,\left(t\right)}{1+\exp\left(\frac{-39.3-V_{\mathrm{cone}}\,\left(t\right)}{4.88}\right)}+u_{\mathrm{resting}}$$

where *u_resting_* = 0.05 to represent the resting calcium concentration in cone terminals (Jackman et al., 2009), following the original hLNS framework paper. Rate constants in the kinetics module also follow the set of formulations for cone synapses in the original paper (He et al., 2022), as


(11) $$\alpha \,\left(u_{\mathrm{cone}}\right)=\left(A_{s}*u_{\mathrm{cone}}^{B_{s}}+C_{s}\right)*\frac{k_{s}^{n_{s}}+u_{\mathrm{cone}}^{n_{s}}}{k_{s}^{n_{s}}}$$

(12) $$\beta \,\left(u_{\mathrm{cone}}\right)=\left(A_{s}*u_{\mathrm{cone}}^{B_{s}}+C_{s}\right)*\frac{k_{s}^{n_{s}}+u_{\mathrm{cone}}^{n_{s}}}{u_{\mathrm{cone}}^{n_{s}}}$$

where *A*_*s*_, *B*_*s*_, *C*_*s*_,*k*_*s*_, and *n*_*s*_ are free parameters in optimization.

To match the experimental measurements, two models for experiments are different after the synaptic block. In the LNS model for off-type bipolar cells, the outputs of the synapse block are converted to the membrane potential by the third non-linearity *N_BC_*, the same as Equation 4 (Figure 2A). In the LNS model for cone synapses, we append a temporal filter (Equation 8 with τ_*L*_ = 60 ms) to mimic the recording method with the glutamate sensor iGluSnFR (Figure 5C; Oesterle et al., 2020).

### Experimental procedures

The stimulus protocol to measure the frequency tunning on frequencies and related experimental recordings are from Grabner et al. (2016). It manipulates the luminance level *x*(*t*) between the dark state (*x*(*t*) = 0, labeled as “off”) and the light state (*x*(*t*) = 1, labeled as “on”) with different temporal frequencies, ranging from 1 to 333 Hz (Figure 1). Initially, the cell (off-type bipolar cell or cone) rests in a dark environment. In a single repeat, the neural activity (membrane potential or synaptic outputs) is depressed in the light and rebounds to a transient response when the light turns off. The amplitude of peak responses relative to the dark baseline decreases with the increment of the temporal frequency. Here, we use the amplitudes as the features of frequency tuning in a visual processing channel. In the experiment of bipolar cells, the peak response is the maximum amplitude of the last repeat, corrected by the resting potentials at dark (Figure 1B). In the experiment of cones, the peak response is the maximum vesicle release rate, corrected by the resting release rate (Figure 6).

The experiment to access the synaptic adaptation follows the original experiment in Grabner et al. (2016). The cone membrane voltage is kept at the resting potential −70 mV initially. Two paired pulses (−30 mV) trigger transient responses of the cone-BC synapse with different time intervals Δ*t*, ranging from 10 ms to 20 s (Figure 3C). The synaptic adaptation depresses the response of the 2nd pulse if the time interval is short. We use the ratio of two peak responses R to indicate the recovery of the synapse from depression. To get the recovery time course τ, pairs of (Δ*t*, R) are used to fit the one-exponential function:


(13) $$R\,\left(\Delta \,t\right)=\mathrm{flu}*\left(1-\exp\left(-\frac{\Delta \,t}{\tau }\right)\right)+\mathrm{base}$$

where *flu* and *base* are free parameters in the curve fitting.

### Model implementation and optimization

We applied all models in the Python2/3 programming environment. We use the “Nelder-Mead” method from the *scipy* package to find the most suitable parameters (Gao and Han, 2012). In the training stage for off-type bipolar cell models, the model’s error is the sum of differences between the feature of simulated responses and experimental recordings. Specifically, features are peak amplitudes and the ratio of peak to the base of membrane voltage at 1-Hz, to control the total amplitude of the curve. In the training stage for cone synapses, the model’s error is the sum of the absolute error of the simulated trace after normalization. All models are set to their stable state before the stimulation begins. The simulation timestep is 1 ms in all cases, and all time constants in models are larger than 1 ms to ensure the correctness of the simulation.

## Data availability statement

Publicly available datasets were analyzed in this study. Datasets can be found in original articles: https://doi.org/10.7554/eLife.67851 and https://doi.org/10.1016/j.neuron.2016.05.019. Codes for data analysis and simulation are available at https://github.com/heliy/Cone-CBC-synapse-parallel-channels.

## Author contributions

LH, LM, and TH contributed to the design and implementation of the research. LH, YH, and LM drafted the manuscript and designed the figures. All authors contributed to the article and approved the submitted version.
